# Novel Electromagnetic Sensors Embedded in Reinforced Concrete Beams for Crack Detection

**DOI:** 10.3390/s19235175

**Published:** 2019-11-26

**Authors:** Michaela Gkantou, Magomed Muradov, George S. Kamaris, Khalid Hashim, William Atherton, Patryk Kot

**Affiliations:** 1 Built Environment and Sustainable Technologies (BEST) Research Institute, Liverpool John Moores University, Liverpool L3 3AF, UK; 2 Sensor City Liverpool Limited, 31 Russell Street, Liverpool L3 5LJ, UK

**Keywords:** concrete beams, crack detection, electromagnetics, non-destructive, sensor

## Abstract

This paper investigates the possibility of applying novel microwave sensors for crack detection in reinforced concrete structures. Initially, a microstrip patch antenna with a split ring resonator (SRR) structure was designed, simulated and fabricated. To evaluate the sensor’s performance, a series of structural tests were carried out and the sensor responses were monitored. Four reinforced concrete (RC) beam specimens, designed according to the European Standards, were tested under three-point bending. The load was applied incrementally to the beams and the static responses were monitored via the use of a load cell, displacement transducers and crack width gauges (Demec studs). In parallel, signal readings from the microwave sensors, which were employed prior to the casting of the concrete and located along the neutral axis at the mid-span of the beam, were recorded at various load increments. The microwave measurements were analysed and compared with those from crack width gauges. A strong linear relationship between the crack propagation and the electromagnetic signal across the full captured spectrum was found, demonstrating the technique’s capability and its potential for further research, offering a reliable, low-cost option for structural health monitoring (SHM).

## 1. Introduction

The maintenance of structures is essential in various industries, including civil, offshore, marine and transportation engineering. The inspection of structures in order to detect structural damage and to assess the condition of materials is an important part of the maintenance process. Crack formation and propagation due to fatigue or earthquake loading, corrosion in metals and delamination in composites can be addressed via effective maintenance [1]. Effective maintenance not only improves the health and safety standards of a structure, but can also allow for cost savings. Often costs related to continuous monitoring, inspection and maintenance can affect civil infrastructure systems and potentially result in decision-making under uncertain environments. This is particularly common in highways and railway bridges, where an over-stretched transport network is self-evident [2]. Highways England alone has an annual budget of over £1 billion allocated to the maintenance of UK’s strategic highways, which make up approximately 10% of the overall UK road network [3]. 

Structural health monitoring (SHM) provides continuous feedback regarding the “state” of the constituent materials and structural members. Continuous monitoring over a period of time offers a full history database for a structure, which can be used to provide a prognosis (evaluation of damage, residual life, etc.) [4]. Deterioration owing to usage, environmental conditions and accidents can be monitored and utilised in order to assess whether the condition of a structure is in accordance with design standard limits and requirements. 

In recent years, different SHM technologies have been investigated aiming to minimise the costs related to damage assessment and maintenance works, while securing the reliability of the structural performance. Traditional sensor technologies (e.g., strain sensors, displacement transducers, accelerometers, etc.) can be utilized, along with traditional visual inspection and damage detection, for structures subjected to various load conditions, such as earthquakes, wind and fatigue loading. In addition to traditional sensors, new measuring approaches (e.g., GPS, terrestrial laser scanning, vision-based systems, etc.) have been applied for structural health monitoring purposes [5]. Non-destructive evaluation (NDE) can be time-consuming and expensive, and access is not always possible [6]. Both wired and wireless sensor technologies are available, each of them having their own advantages and limitations, while the main concern should always be obtaining high accuracy data. Existing wired sensor techniques can be expensive in terms of additional costs and the space required for cable installation, while wireless sensors require due consideration for the power supply.

Aiming to complement existing sensing technologies for SHM, the present study investigates the potential of using microwave sensors for crack detection in concrete structures. Recently, electromagnetic sensors have received interest as a method to monitor and predict the excess moisture content in concrete structures due to exposure [7,8,9]. The use of microwave technology offers low cost, low profile flexibility in the design and the adaptation of sensors. This study will demonstrate a potential sensor solution for the detection and prediction of crack propagation in reinforced concrete (RC) beams. Note that wired sensors are examined herein, whereas wireless microwave sensor technologies are planned to be studied in a future research programme, in which energy harvesting systems will be considered as a power source for the sensors. 

## 2. Design of the Experiments

An experimental programme was designed and carried out in order to investigate the accuracy of the microwave sensing technology in predicting the crack formation in reinforced concrete beams. The current section provides details regarding the design of the RC beams (Section 2.1) and of the microwave sensors (Section 2.2).

### 2.1. Design of the Beams

**Figure 1:**
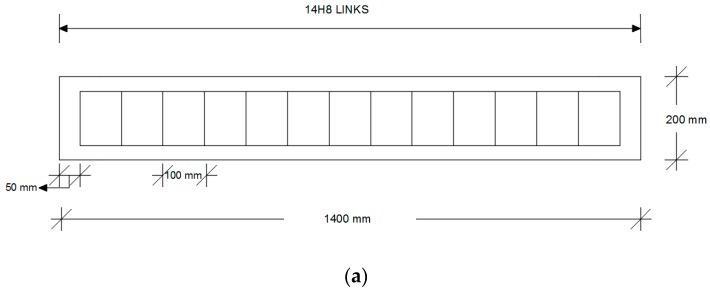

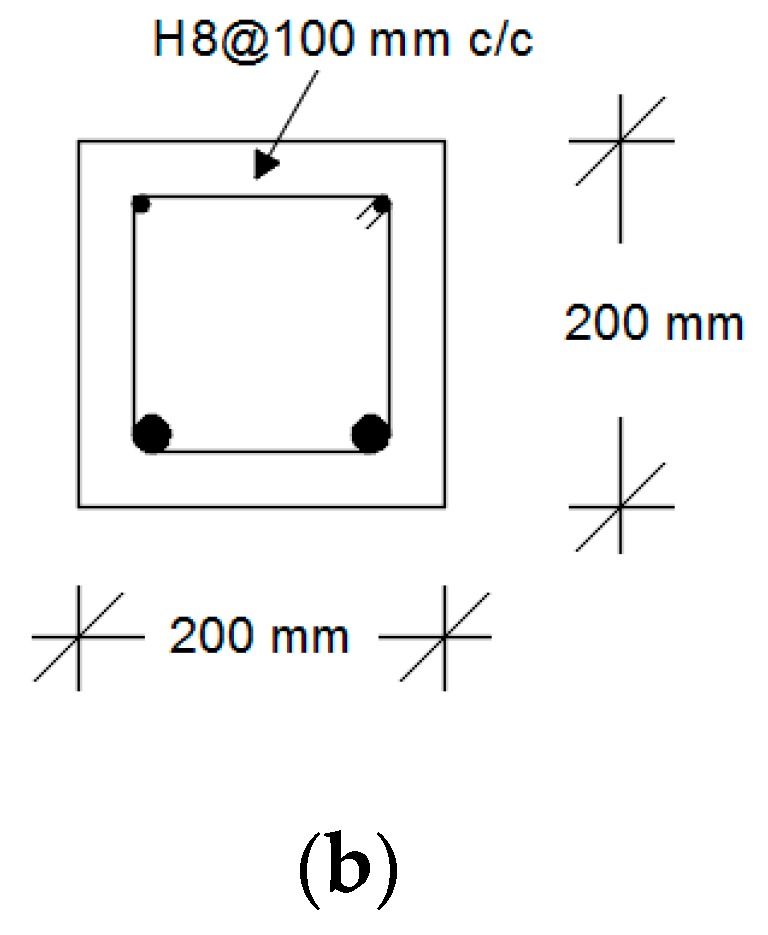
Design details of the beams used for the experimental set-up in this study: (**a**) Longitudinal and shear reinforcement; (**b**) Cross-section of the beam.

Four RC beams were designed in accordance with Eurocode 2 [10] to be used for the experimental set-up of this study. The total length of the beams was 1400 mm and their cross-section was rectangular with dimensions of 200 mm × 200 mm. The beams were subjected to a vertical design load equal to a maximum of 100 kN based on the capacity of the actuator and associated load cell. Grade C40/50 was used for the concrete and steel grade S500 (Scimitar Steels, Liverpool, UK) for the reinforcement. The cover to the reinforcement for bond and durability was 30 mm. Steel bars with diameters of 20 mm and 8 mm were used for the longitudinal reinforcement and the hanger bars of the beams, respectively. Finally, shear links (Scimitar Steels, Liverpool, UK) with diameter 8 mm with 100 mm spacing’s were used for the shear reinforcement of the beams. The design of the beams is shown in Figure 1. The microwave sensor (Liverpool John Moores University, Liverpool, UK) was placed 100 mm from the bottom of the concrete specimen in order to capture the crack propagation in the tensile zone just below the neutral axis. 

### 2.2. Design of the Microwave Sensors

In this study, a microstrip patch antenna with a split ring resonator (SRR) structure was designed, simulated and fabricated as a potential microwave sensing technique. SRR structures have been utilised to realise microwave resonators, filters and microwave antennas [11], and have been used for various applications, namely chemical sensors [12], gas sensors [13], crack detection sensors [14], angle sensors [14], alignment and position sensors [15], displacement sensors [16] and microwave imaging sensors [17]. 

**Figure 2:**
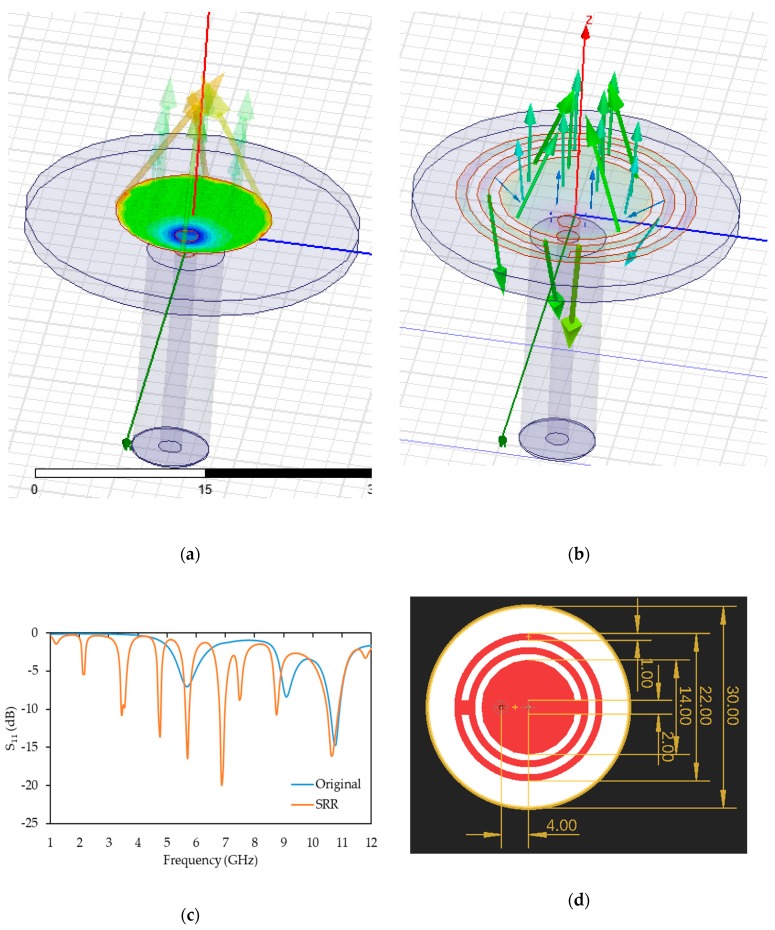
Simulated (**a**) original antenna, (**b**) antenna with split ring resonators (SRRs), (**c**) the S_11_ simulation results and (**d**) the design of the proposed antenna with dimensions (mm).

Figure 2 presents (a) a traditional circular patch antenna resonating at 10.8 GHz (30 mm in diameter) and (b) a traditional circular patch antenna with an additional SRR structure, which were modelled and simulated using the High Frequency Structural Simulation software tool (HFSS) (ANSYS Inc., Canonsburg, PA, USA) [18]. SRR structures offer an improved performance compared to traditionally designed microstrip patch antennas as they allow for miniaturised antenna dimensions and/or multiband operations, i.e., multiple resonant frequencies [19]. In this study, the SRR structure was used to provide multiple resonant frequencies between 1 to 10 GHz, which provided more details about the concrete. In addition, the use of SRR shifted the resonant frequencies to lower frequency ranges, which in turn increased the penetration capability of the antenna, while preventing disturbances in the concrete structure by keeping the dimensions of the antenna small. Figure 2c demonstrates the comparison between the resonant frequency response of the traditional circular patch antenna (Figure 2a) and the proposed SRR structure (Figure 2b), which resonated at multiple frequencies below 10.8 GHz, namely 3.4 GHz, 4.7 GHz, 5.7 GHz, 8.7 GHz and 10.6 GHz with a return loss below −10 dB. The dimensions (in mm) of the proposed antenna are presented in Figure 2d.

**Figure 3:**
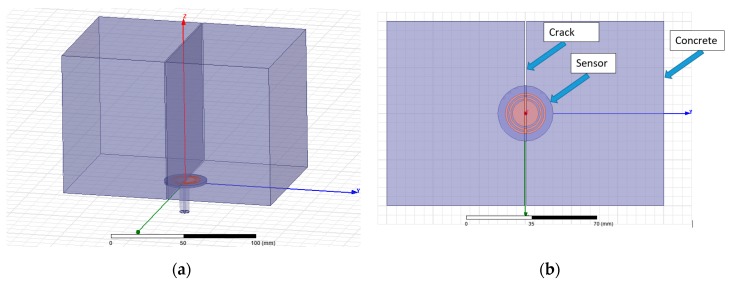
HFSS simulation of the proposed antenna and the concrete sample: (**a**) 3D view and (**b**) top view.

As the sensor was aimed to be embedded into the concrete, a concrete sample was modelled and simulated using HFSS. According to Piladaeng [20], the concrete dielectric constant for a 7-day-old specimen is between ε′ = 4 and ε′ = 5.5, conductivity (S/m) in the range between 0.18 and 0.25 and dielectric loss factor between ε″ = 0.45 and ε″ = 0.65. Therefore, in this study, the concrete specimen was modelled using ε′ = 5.5, conductivity (S/m) of 0.25 and a loss factor of ε″ = 0.5. The model of the concrete sample was simulated with various crack widths (0.0 mm, 0.1 mm, 0.2 mm, 0.3 mm, 0.4 mm, 0.5 mm and 0.6 mm) and with the SRR antenna underneath the sample (see Figure 3). The simulation was carried out seven times, including the case of an uncracked concrete sample. The simulation measurements (S_11_, reflection coefficient) were realised in the frequency range of 1 to 10 GHz and with 4000 sweep points. 

**Figure 4:**
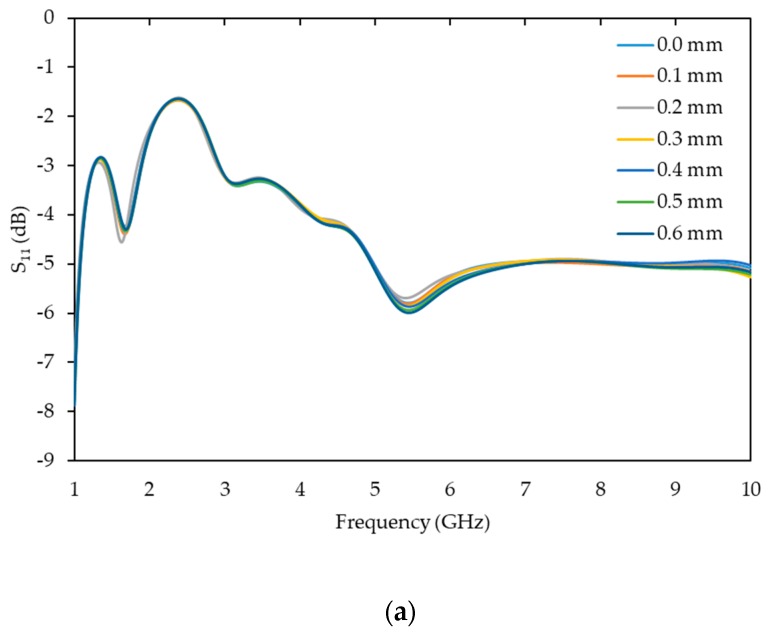

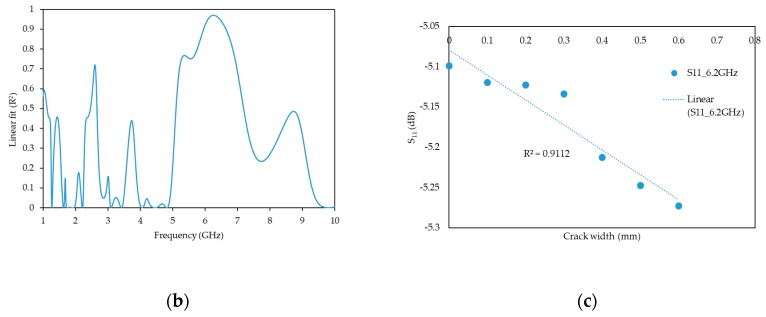
Simulated (**a**) raw data, (**b**) R^2^ values across the full spectrum (1–10 GHz) and (**c**) linear fit between the S_11_ change and crack width at 6.2 GHz, with R^2^ = 0.91.

The simulation results are shown in Figure 4a, which mainly demonstrates a change in the amplitude of the reflected signal (S_11_). The full simulated frequency spectrum (1–10 GHz, 4000 sweep points) was scanned across using a bespoke LabVIEW (v2013) program (National Instruments, Austin, TX, USA) aiming to identify a linear relationship between the sensor response (S_11_) and the crack width (mm). Figure 4b presents the linear fit across the full simulated spectrum and the crack width (mm). The strongest linear correlation can be observed around 6.2 GHz. Figure 4c shows the linear relationship between S_11_ and the crack width (mm) changed at 6.2 GHz, with R^2^ = 0.91. The figure demonstrates that S_11_ increased as the crack width grew, i.e., less energy/power was absorbed by the concrete sample as there was an opening air gap, which changed the penetration of the electromagnetic (EM) signal, as well as changing the reflection of the signal. Based on these simulation results and previous work [8], the investigation was taken forward, i.e., to the fabrication of the sensors and subsequently to the experimental testing. 

**Figure 5:**
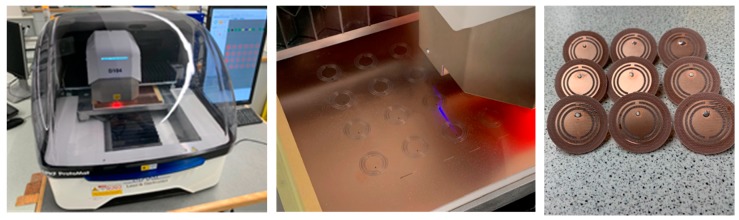
Fabrication of the antennas on a LPKF ProtoMat D104.

The proposed antennas were fabricated on a double-sided 1.6 mm FR4 PCB (Printed Circuit Board) sheet using an LPKF ProtoMat D104 unit (see Figure 5). Then the SMA connectors were inserted through the probe-fed hole of the antennas and soldered on the patch (top layer) and the ground plane (bottom layer) of the antennas.

## 3. Experimental Programme

In order to evaluate the capability of the microwave sensors to predict the crack formation, four reinforced concrete beams, in line with the design described in Section 2.1, were tested under three-point loading. The experimental programme was carried out at the Henry Cotton Laboratory of Liverpool John Moores University. The preparation of the specimens, including the attached instrumentation, is described in Section 3.1, while Section 3.2 provides information on the test set-up and the process utilised.

### 3.1. Specimens’ Preparation and Instrumentation

Initially, the formwork for the concrete beams was constructed and the longitudinal steel reinforcement bars and the shear links were fabricated. Upon careful treatment of the surfaces, six strain gauges (Techni Measure - Sensors & Transducers, Doncaster, UK) were attached at the tensile reinforcement bars of the specimens. In particular, the strain gauges were adhered to the bottom and the top of a tensile reinforcement bars and at the mid and quarter span. A microwave sensor was located at the neutral axis (NA) of the cross-section mid-span of each specimen, where negligible longitudinal stresses and strains were expected. 

Following this, the concrete mix was prepared in accordance with the BRE (Building Research Establishment) concrete mix design [21], which gave a 28-day compressive strength equal to 57 N/mm^2^. Crushed limestone aggregates (Beers Timber and Building Suppliers, Liverpool, UK) (20 mm down), sand (Mersey Grit) (Beers Timber and Building Suppliers, Liverpool, UK), OPC Type II (Travis Perkins, Liverpool, UK) and a water-to-cement ratio equal to 0.45 were used. A slump test was also performed to ensure that the mix was in accordance with the design guidelines. The beams were cast and the concrete was vibrated using a poker vibrator according to the design guidelines [21]. Note that the microwave sensor was embedded into concrete and the accuracy of its location was regularly checked during casting.

Concrete cubes with dimensions of 150 × 150 × 150 mm were also prepared in order to check the compressive strength of the concrete at 7 and 28 days. 

**Figure 6:**
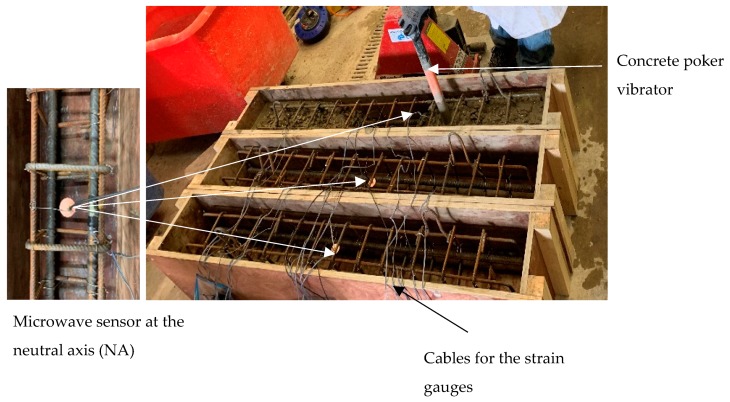
Specimens’ preparation.

The specimens’ preparation, including the details of the attached sensors and gauges along with the concrete casting, are shown in Figure 6. 

Upon demoulding, the beams were painted on both lateral sides in order to make the crack formation and propagation detectable. Demec studs were applied at the central tension region of the beams in order to monitor the crack opening within the most heavily stressed areas. Four stud sets were bonded on the concrete’s surface at a set horizontal distance of 50 mm on either side of the centreline and at vertical distances equal to 80 mm, 60 mm, 40 mm and 20 mm from the beam’s neutral axis (NA).

### 3.2. Experimental Set-Up

**Figure 7:**
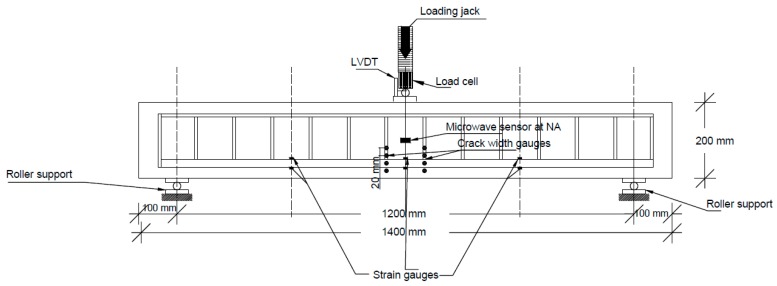
Experimental set-up.

**Figure 8:**
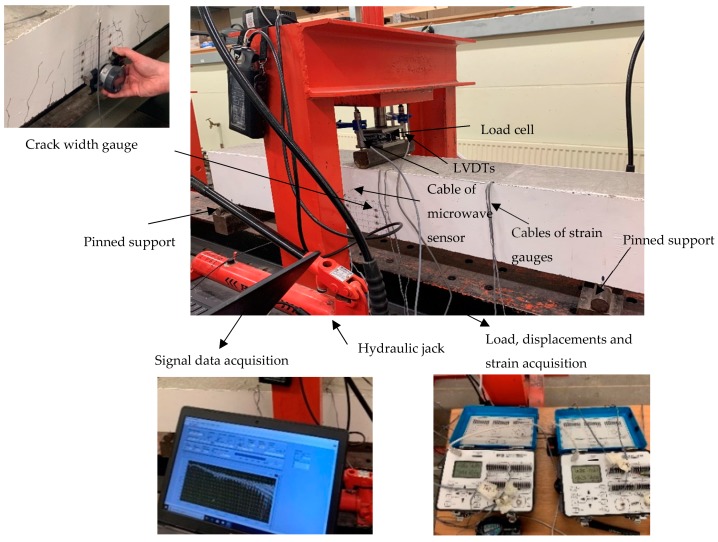
Photos from the experimental set-up and instrumentation. LVDTs: ???

Having attached the required instrumentation (i.e., strain gauges attached on the tensile reinforcement bars, microwave sensors embedded in the concrete beam and Demec studs glued on the concrete beam’s surface), the specimens were cured for 7 days and were then located at the test rig for the experimental investigation. The beams spanned 1200 mm with an overhang of 100 mm from both sides of the supports. Simply-supported conditions were achieved with the use of roller supports. A calibrated hydraulic 100 kN load actuator was used for the load application. A constant slow loading rate, excluding any dynamic effects, was implemented, while a load cell was used for the recording of the load increments. Two strain gauge type displacement transducers (DTs) were placed at the mid-span in order to measure the vertical deflections of the specimens during testing. Synchronised data acquisition systems were used for the structural response measurements. The experimental set-up, along with the employed instrumentation, are shown in Figure 7 and Figure 8. The cables shown in Figure 8 present the current available instrumentation for the structural response monitoring via strain gauges, Linear Variable Displacement Transducers (LVDTs) and the load cell. The microwave sensor in this study was connected using a single coaxial cable, which will be eliminated in the future.

## 4. Results

### 4.1. Results on the Structural Response

**Figure 9:**
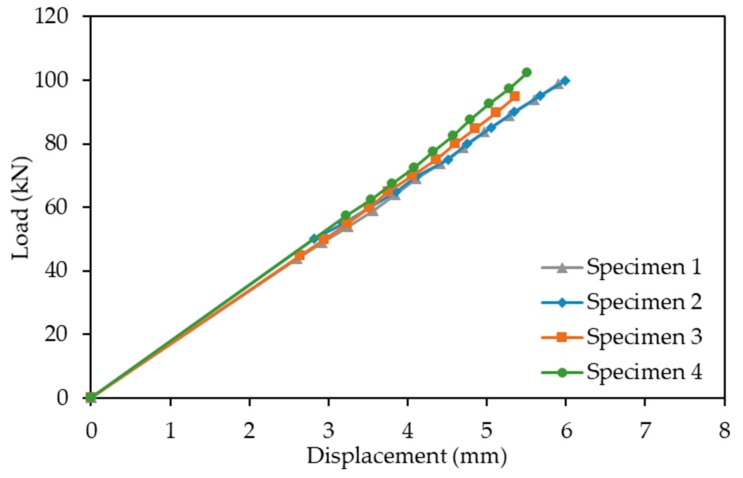
Load versus average mid-span displacement graph of all test specimens.

**Figure 10:**
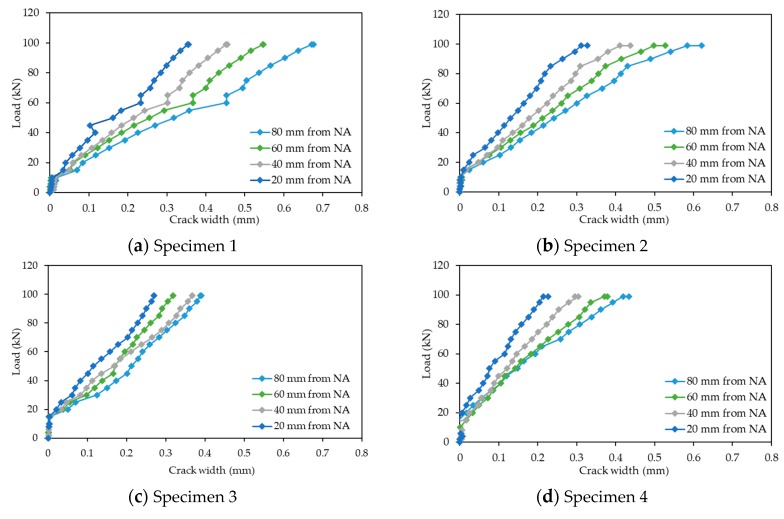
Load versus crack width opening.

The structural response for all specimens is shown in Figure 9, where the applied load is plotted against the average of the two DTs for the mid-span displacement. As shown, the load versus vertical displacement response of the beams were linear, and very similar for all specimens. The crack width recordings for the specimens are presented in Figure 10, where in all cases, the crack opening increased for higher loads. Additionally, the observed crack width openings were more pronounced for a vertical distance of 80 mm from the neutral axis, where the tensile stresses were higher, compared to those of smaller vertical distances from the neutral axis. The strain gauges measurements were only used to further validate the structural response of the beam and will not be further utilised in this paper. 

**Figure 11:**
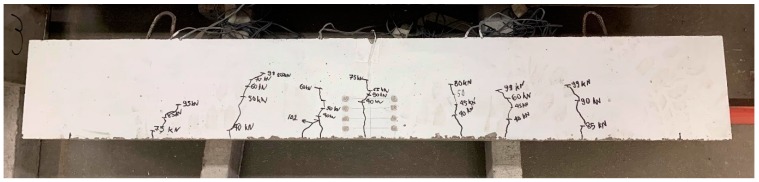
Typical cracked specimen at the maximum applied load.

For each of the specimens, a concrete cube compressive strength test was performed. The average compressive strength was 49.92 N/mm^2^ and 57.15 N/mm^2^ for the 7- and 28-day tests, respectively. In Figure 11, a typical cracked specimen at the end of the testing is depicted. The crack evolution and propagation was marked during testing. A symmetrical crack pattern, as anticipated for reinforced concrete beams under pure flexure, was observed in most cases. It is noteworthy that even though the specimens did not reach their failure load, the recorded response up to 100 kN allowed for the comparison between the Demec studs and the new sensor signal values, thus serving the scope of this study, as will be discussed in Section 4.2.

### 4.2. Results for the Microwave Sensor

**Figure 12:**
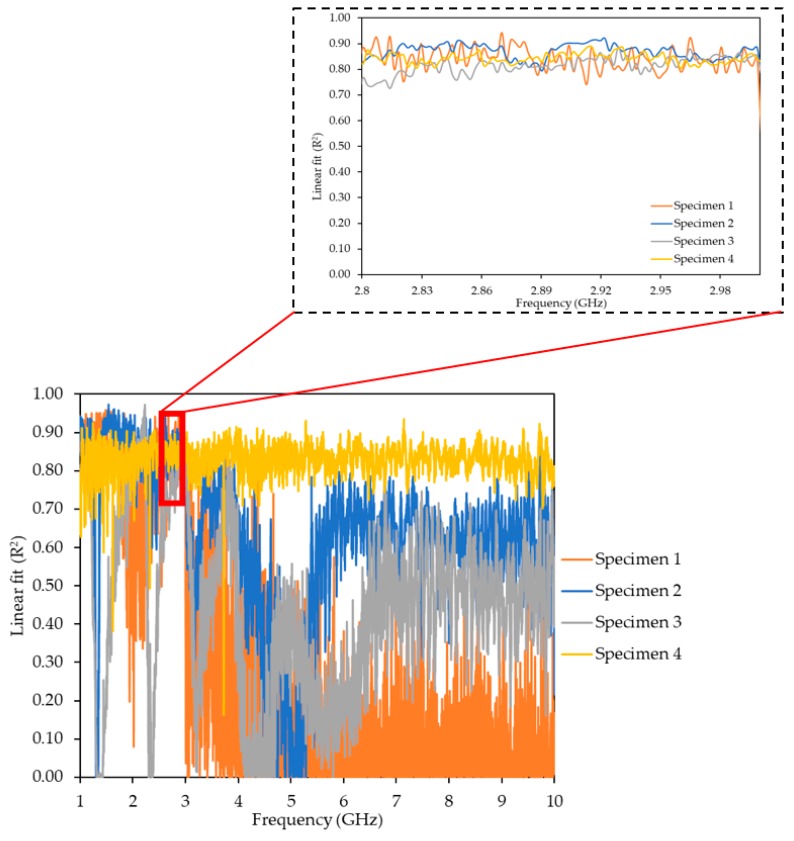
Linear fit between the crack width and electromagnetic (EM) response for all four specimens across the 1–10 GHz frequency spectrum.

The microwave measurements were provided by the S-parameter (i.e., S_11_, reflection coefficient), namely complex data (real and imaginary data of the signal) and magnitude from the Vector Network Analyser (VNA) (Rohde & Schwarz, Munich, Germany). The data was captured via a bespoke LabVIEW program on a laptop connected to the VNA. The data acquisition took place at each load increment. The recorded measurements were in the 1 to 10 GHz frequency range using the maximum number of sweep points available (4000). The captured results were analysed against the crack width of the samples in order to determine a relationship between the changes in the EM response and the crack width. Figure 12 demonstrates a linear relationship between the crack propagation and the EM signal across the fully captured spectrum (1–10 GHz). The strongest linear relationship was identified between the 2.8 GHz and 3 GHz frequency range. 

**Figure 13:**
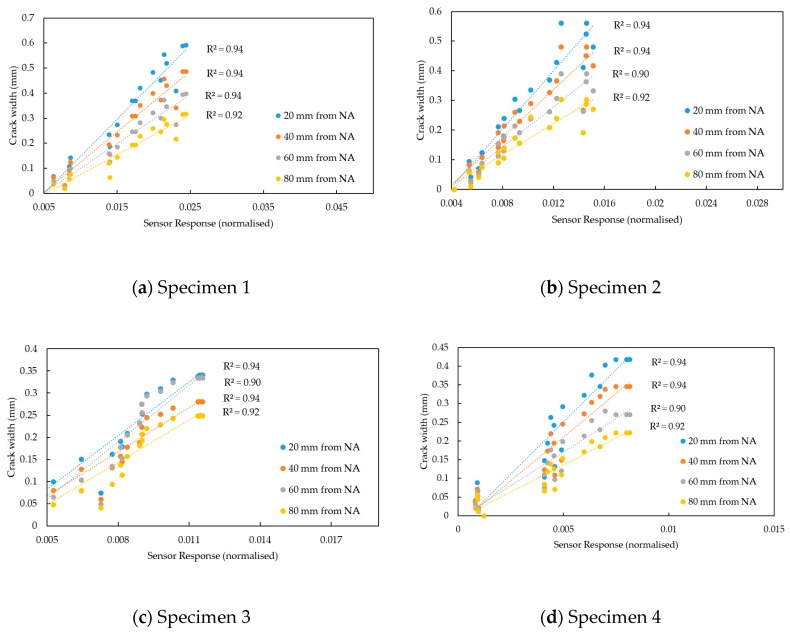
Linear correlation between the crack width and sensor response at 2.9 GHz.

**Figure 14:**
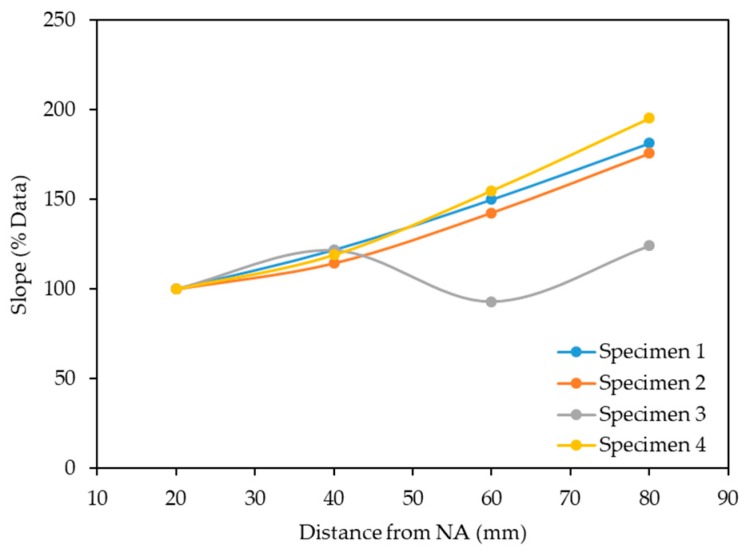
Slopes of linear regression lines for four specimens at 20 mm, 40 mm, 60 mm and 80 mm distances from the NA.

The EM response from all four specimens demonstrated a strong linear relationship against the crack width with R^2^ above 0.90 for specimens 1, 2, 3 and 4 (Figure 13). The slopes of the linear regression lines for the four specimens at 20 mm, 40 mm, 60 mm and 80 mm distances from NA (Figure 14) demonstrate a similar response for specimens 1, 2 and 4, namely the slope increased with the increase of the distance from the NA. For specimen 3, the crack width at 20 mm and 60 mm distances from NA were found to be similar (Figure 13c), leading to a slope decrease from 40 mm to 60 mm, as shown in Figure 14. In addition, the sensor response varied between the specimens owing to the inconsistency of the crack patterns on each specimen. Note also that only small changes (crack widths less than 1 mm) were considered for this study. Nevertheless, the sensor was able to determine these small changes in the structure, i.e., it demonstrated a potential to detect anomalies in RC structures, which could be used as an indicator of a structural failure. Further study is required for the proposed technique, namely more samples with various crack widths and patterns in order to obtain necessary parameters/data for the analysis or the potential implementation of machine learning algorithms. 

## 5. Conclusions

Aiming to assess microwave sensing technology as a means of structural health monitoring, four reinforced concrete beams were tested under three-point loading in the Henry Cotton Laboratory of Liverpool John Moores University. In addition to the commonly implemented instrumentation in structural testing (i.e., load cells, displacement transducers, Demec studs, etc.), a novel microwave sensor, specifically designed for this application, was inserted into each beam’s reinforcement cage before the concrete’s casting. In order to allow for a reduced size of the sensor and to shift the resonant frequencies to lower frequency ranges, hence increasing the antenna’s penetration capability, a microstrip patch antenna with an SRR structure was designed and subsequently fabricated. 

During the load application, both the structural performance and the signal measurements were incrementally recorded. As anticipated, a linear load–displacement structural response was found to be very similar for all specimens. The microwave data sets, which were captured via a bespoke LabVIEW program, were provided by the S_11_ reflection coefficient, and were within the 1–10 GHz frequency range. For the largest anticipated tensile crack at the mid-span of the beam, the readings for the crack width gauges and the sensors were processed and compared. A strong linear relationship with R^2^ values very close to unity were observed. The variance on the sensor response was related to the different crack patterns observed in each specimen. The present study considered crack widths below 1 mm and thus only small changes in the concrete structure. It is noteworthy that the sensor was able to detect these small changes in the concrete’s structure, demonstrating that the proposed technique could be further studied in order to detect cracking in RC structures. Further investigation is required to obtain more data for various crack patterns and widths, thus enhancing the measurements’ repeatability and reliability and allowing for the technique’s validation.

Future studies could also investigate the possibility of insulating/sealing the antennas to avoid direct contact with the concrete, which might change the properties of the antenna owing to the physical and chemical properties of the concrete mixture. The extra layer could protect antennas from possible corrosion/oxidation of the copper part of the antenna. Finally, additional sensors (i.e., moisture and temperature) could be added to provide additional variables for the sensor algorithm, which could further improve crack detection.
